# Successful endovascular therapy for iatrogenic vertebral artery injury using intravascular imaging

**DOI:** 10.1093/ehjcr/ytag493

**Published:** 2026-07-10

**Authors:** Shogo Matsui, Miwa Arakawa, Noboru Oda, Masaya Kato

**Affiliations:** Department of Cardiovascular Medicine, Hiroshima City North Medical Center Asa Citizens Hospital, 1-2-1 Kameyama, Asakita-ku, Hiroshima 731-0232, Japan; Department of Cardiovascular Surgery, Hiroshima City North Medical Center Asa Citizens Hospital, Hiroshima 731-0232, Japan; Department of Cardiovascular Medicine, Hiroshima City North Medical Center Asa Citizens Hospital, 1-2-1 Kameyama, Asakita-ku, Hiroshima 731-0232, Japan; Department of Cardiovascular Medicine, Hiroshima City North Medical Center Asa Citizens Hospital, 1-2-1 Kameyama, Asakita-ku, Hiroshima 731-0232, Japan

## Case description

**Figure 1:**
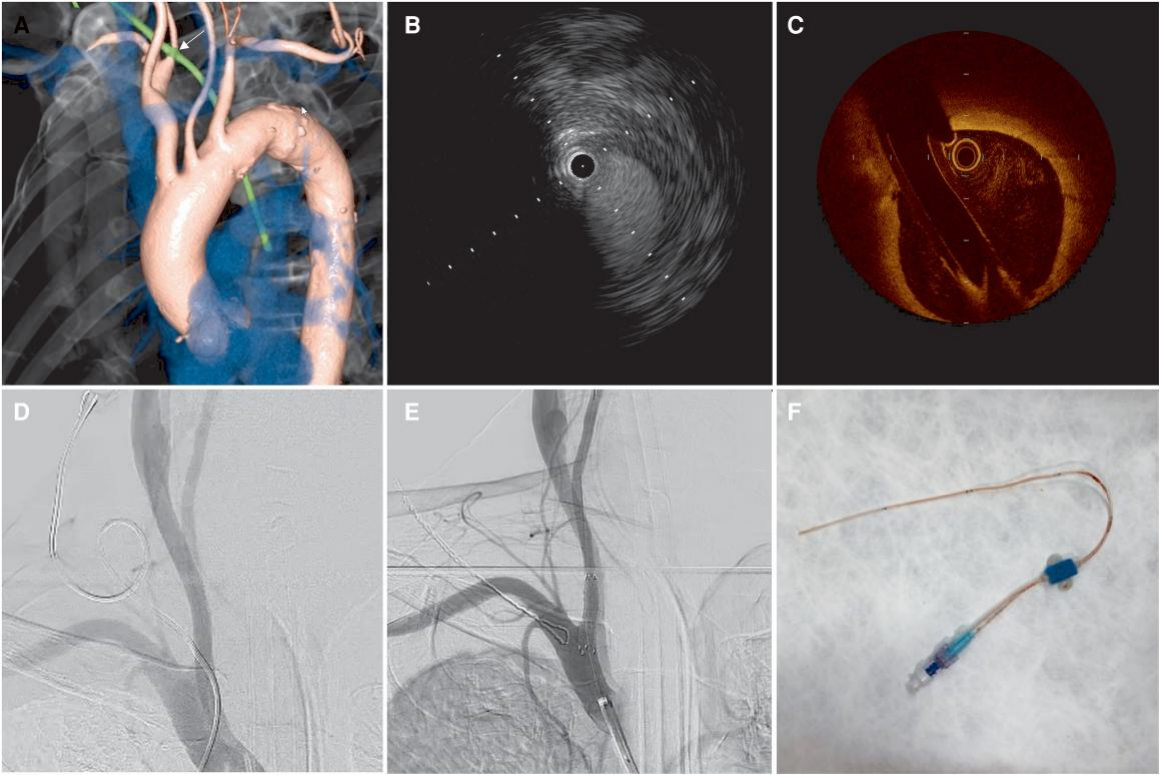
Endovascular therapy for iatrogenic vertebral artery injury using multimodality intravascular imaging. (*A*) Computed tomography revealed the possibility that the central venous catheter had penetrated the right vertebral artery. (*B*) Intravascular ultrasound showed an unusual attenuation in the vertebral artery. (*C*, and Supplementary material online, *Video S1*) OCT clearly demonstrated a tubular structure penetrating the vertebral artery. (*D–F*, and Supplementary material online, *Video S2*) The VIABAHN stent graft was successfully implanted at vertebral artery after removal of the central venous catheter.

An 88-year-old man was admitted with aspiration pneumonia. Insertion of a catheter was attempted via the right internal jugular venous route. However, a subsequent computed tomography angiography revealed the possibility that the central venous catheter (CVC) had accidentally penetrated the right vertebral artery (*Figure 1A*, arrow = CVC).

Given the intrathoracic location of the penetration site, surgical repair was considered too invasive. In addition, four-vessel cerebral angiography demonstrated hypoplasia of the right vertebral artery without collateral communication to the anterior circulation, indicating that coil embolization of the right vertebral artery was not feasible. Intravascular ultrasound showed an unusual attenuation at the proximal site of vertebral artery (*Figure 1B*). Optical coherence tomography (OCT) clearly demonstrated a tubular structure penetrating the vertebral artery (*Figure 1C*). OCT findings indicated that CVC removal without vascular protection has a risk of fatal intrathoracic haemorrhage. Therefore, a covered stent strategy was selected as a minimally invasive and reliable treatment. The VIABAHN stent graft was deployed at the vertebral artery concurrently with the removal of the CVC (*Figure 1D-F*). Following stent graft implantation, dual antiplatelet therapywith aspirin and clopidogrel was administered for 6 months, after which clopidogrel was discontinued. Computed tomography angiography performed the following day revealed stent graft patency without haemorrhagic complications. Post-operatively, the patient was discharged without any neurological or bleeding complications.

Insertion of CVC still has a potential risk of arterial injury, despite the development of ultrasound-guided puncture.^1^ We performed successful endovascular therapy for iatrogenic VA injury using multimodality intravascular imaging. OCT may be useful for detecting intravascular foreign bodies, including iatrogenic artery injury caused by the CVC.

## Supplementary Material

ytag493_Supplementary_Data

## Data Availability

The data underlying this article are available upon reasonable request to the corresponding author.

## Notes

**Consent:** Written informed consent for publication of this case report including images was obtained from the patient.

## References

[1] Kang H, Cho SY, Suk EH, Ju W, Choi JY. Massive hemothorax following internal jugular vein catheterization under ultrasound guidance: a case report. World J Clin Cases 2022;10:5776–5782. 35979121 10.12998/wjcc.v10.i17.5776 PMC9258369

