# Supplementary material for: Successful endovascular therapy for iatrogenic vertebral artery injury using intravascular imaging
Source: Eur Heart J Case Rep. 2026 Jul 10;10(7):ytag493. doi: 10.1093/ehjcr/ytag493 (PMC13412386; doi:10.1093/ehjcr/ytag493)
Supplement: ytag493_Supplementary_Data [file ytag493_supplementary_data.zip › Supplementary video captions.docx]

**Video Legends**

**Online Video 1. Optical coherence tomography of the vertebral artery.**

Optical coherence tomography clearly demonstrated a tubular structure penetrating the vertebral artery.

**Online Video 2. Angiography of the endovascular therapy.**

Angiography shows controlled withdrawal of the central venous catheter with simultaneous deployment of a covered stent across the injured vertebral artery segment.
